# Cervical Lymphadenopathy—Pitfalls of Blind Antitubercular Treatment

**Published:** 2014-03

**Authors:** Sudipta Pandit, Sabyasachi Choudhury, Anirban Das, Sibes Kumar Das, Soumya Bhattacharya

**Affiliations:** ^1^Department of Pulmonary Medicine, Medical College, Kolkata, India;; ^2^Department of Chest Medicine, Bankura Sammilani Medical College, Bankura, India

**Keywords:** Cervical lymphadenopathy, Excision biopsy, Hodgkin disease, Kikuchi's disease, Non-Hodgkin lymphoma, Tuberculosis

## Abstract

Tuberculosis (TB) is the most common cause of cervical lymphadenopathy in the TB-endemic zone, like India but it can also mimic other diseases. Four cases of cervical lymphadenopathy presented to us as initial treatment failure after completion of six months of antitubercular drugs (ATD), including rifampicin, isoniazid, pyrazinamide, and ethambutol. All were diagnosed as having tuberculosis either by fine needle aspiration cytology or clinically from outside our institution. In one case, tuberculosis was the final diagnosis but, unfortunately, it was multidrug-resistant. In other three cases, Hodgkin disease, Non-Hodgkin lymphoma, and Kikuchi's disease were the diagnoses. In resource-poor countries, like India, which is also a TB-endemic zone, TB should be the first diagnosis in all cases of chronic cervical lymphadenopathy, based on clinical and/or cytological evidences. So, they were correctly advised antitubercular therapy (ATT) initially. Sometimes, TB mimics other aetiologies where apparent initial improvement with ATT finally results in treatment failure. Hence, investigations for microbiological and histopathological diagnosis are warranted, depending on the resources and feasibility. If these tests are not routinely available, the patients should be under close monitoring so that lymphoma, drug-resistant TB, or other aetiologies of cervical lymphadenopathy are not missed. Patients with cervical lymphadenopathy rarely presents acutely; so, a physician can take the opportunity of histopathological study of lymphnode tissue.

## INTRODUCTION

In resource-poor countries, like India, which is located in tuberculosis-endemic zone, all cases of cervical lymphadenopathy are traditionally considered a tuberculous aetiology, unless proved otherwise, as tubercle bacilli are the most common offending agent of cervical lymphadenopathy (1). Clinical features, especially constitutional symptoms, local signs, like matting or discharging sinus, pulmonary involvement, background of immune-suppression, like diabetes or human immunodeficiency virus (HIV) infection, strong positive tuberculin skin test, all help clinch the correct diagnosis in almost all cases of tuberculous lymphadenopathy. However, it should be kept in mind that there are many other aetiologies of cervical lymphadenopathy (although rarer than TB), especially in cases of non-responders and where inconclusive cytological picture in absence of strong supportive clinical evidences of TB is the basis of initial advice of antitubercular drugs. In such cases, close monitoring of the patients is mandatory and excision biopsy must be done before starting ATT, if facilities are available. Otherwise, this type of invasive procedure may be avoided in the first visit. Lymphoma and HIV infection should be ruled out in inconclusive cases, and drug resistance and paradoxical reaction, in non-responders. In one of our four cases, Kikuchi-Fujimato disease, a very rare cause of cervical lymphadenopathy was diagnosed on the basis of histopathological examination. In another case, multidrug-resistant *Mycobacterium tuberculosis* complex was isolated from cervical lymphnode culture. Hodgkin disease and non-Hodgkin lymphoma were the other two diagnoses. It is difficult to differentiate TB and lymphoma on clinical ground only, especially when fine needle aspiration cytology (FNAC) is totally inconclusive. On the other hand, granulomas may be found in both TB and lymphoma, especially in cases of Hodgkin disease. In pulmonary tuberculosis, there is option for sputum examination for acid fast bacilli and mycobacterial culture but, in the extra-pulmonary cases, like lymphnode tuberculosis, microbiological proof is difficult to obtain. So, in cytologically-inconclusive cases, and in non-responders, histopathology and mycobacterial culture of biopsy specimen should be tried initially, at the beginning of ATT. GeneXpert test of lymphnode biopsy material is another good option for both microbiological diagnosis and detection of drug resistance (2).

## CASE SERIES

### Case 1

**Figure 1.**
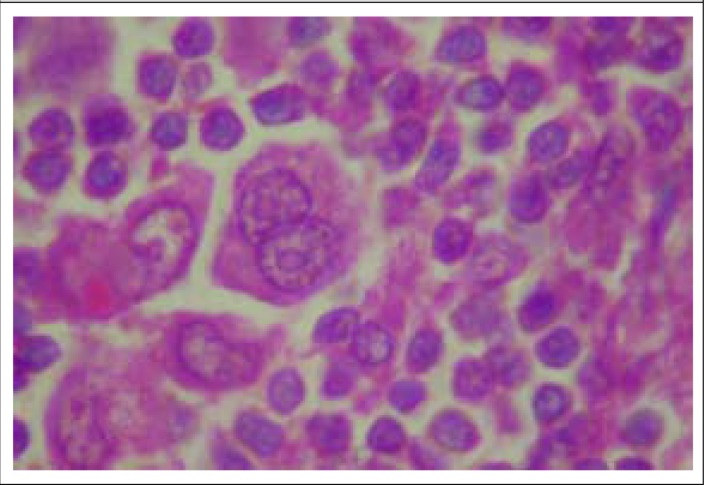
Histopathological picture of left cervical lymph node showing evidence of Hodgkin disease (H&E stain, ×1000)

A young female patient was admitted with bilateral progressive neck swelling and low-grade intermittent fever for 1 year with productive cough for 2 weeks. Because of granulomatous lesion in FNAC of left cervical lymph node, she was diagnosed outside as tubercular lymphadenopathy and took antitubercular drugs (ATD) for 9 months with isoniazid (H), rifampicin (R), ethambutol (E), and pyrazinamide (Z). She had no significant past history. Patient had pallor and jaundice. Cervical, axillary, inguinal and epitrochlear lymph nodes were palpable. Chest x-ray revealed left-sided pleural effusion and rounded opacity in right-middle zone, with mediastinal widening. Initially, the patient had leukocytosis with neutrophilia and normal serum prolactin level. Despite receiving injectable piperacillin-tazobactum for 7 days, leukocytosis persisted. Other routine blood examinations were normal, and 2 samples of sputum smear microscopy for acid fast bacillus (AFB) were negative. FNAC of the cervical lymph node again showed granulomatous lymphadenitis. Contrast Enhanced Computer Tomography (CECT) thorax showed irregular opacity in the right-middle lobe with mediastinal lymphadenopathy with left-sided pleural effusion and pericardial effusion. CT-guided FNAC of right-middle lobe lesion revealed chronic inflammation. Echocardiogram showed large pericardial effusion. CT scan of abdomen showed hepatomegaly, ascites and retroperitoneal lymphadenopathy, and erosion of 12^th^ thoracic vertebra. Pleural fluid was exudative, lymphocytic with high adenosine de-aminase level. Human immunodeficiency virus (HIV) antibody and connective tissue profile were negative. Excision biopsy of the left cervical lymph node revealed replacement of normal architecture by sheets of cells with pleomorphic appearance—lymphocytes, histiocytes, plasma cells, and eosinophils. Mononuclear cells with prominent eosinophilic nucleoli (Hodgkin cell) were present. Histopathology was compatible with Hodgkin lymphoma or Hodgkin disease (HD) (Figure 1). Immunohistochemistry revealed tumor cell expression of CD30, CD15, CD20, Pax5, and immunonegative for LCA, CD3 in a T cell-rich background, which confirms the diagnosis of classical HD. Bone marrow study was normal. The patient was treated with doxorubicin, bleomycin, vinblastine, and dacarbazine. She was discharged in a favourable condition.

### Case 2

**Figure 2.**
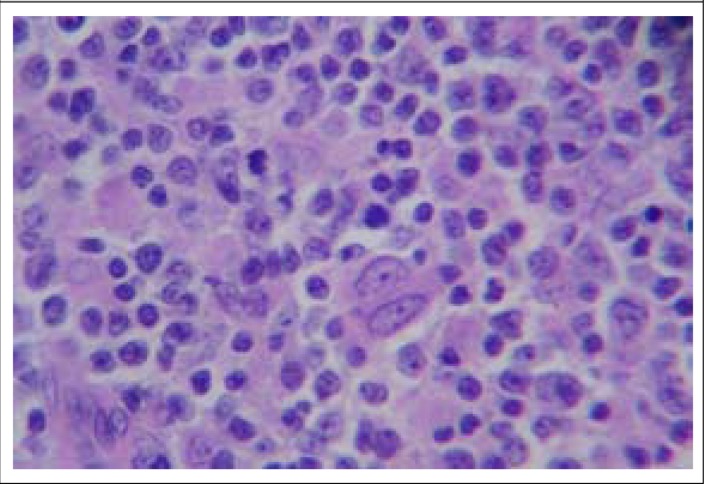
Histopathological picture of left cervical lymph node showing evidence of non-Hodgkin lymphoma (H&E stain, ×400)

A middle-aged male (smoker) was admitted with the complaint of low-grade, intermittent fever with swelling of the neck for 8 months with intermittent productive cough for 10 months. Swelling was bilateral, gradually progressive in nature and left-sided swelling was painful. Clinical examination did not reveal anything significant. FNAC of the left-sided swelling was done elsewhere 6 months back and was diagnosed as tuberculous lymphadenitis with presence of AFB in the FNAC tissue. He took 2 months’ HREZ, followed by 4 months’ HR but the size of the swelling was increasing in nature. Sputum for Z-N stain was negative for AFB throughout the period of 6 months. Chest x-ray and CECT thorax did not reveal any abnormality. Routine blood examinations showed leukocytosis with neutrophilia, and she received injectable co-amoxyclav for 7 days. Gradually, total leukocyte count became normal. Other routine blood examinations were normal, and sputum Ziehl–Neelsen (Z-N) stain was negative for AFB. HIV, HbsAg, and HCV were negative. Excision biopsy of the right cervical lymph node revealed replacement of normal architecture by sheets of atypical large lymphoid cells admixed with histiocytes and plasmacytoid cells. Scattered immunoblasts were present. Histopathology was compatible with NHL (Figure 2). Immunohistochemistry revealed tumour cell expression of CD20, CD10, and immunonegative for CD3, CD5, CD23, and CD30. It was NHL of diffuse large B cell phenotype. After other necessary examinations, the patient was treated with cyclophosphamide, doxorubicin, vincristine, and prednisone. He was discharged in a favourable condition.

### Case 3

A 15-year female student was admitted with the bilateral progressive neck swelling for 1 year, low-grade intermittent fever, and productive cough for 2 weeks. She was diagnosed elsewhere as tubercular lymphadenitis by FNAC. Initially, she was given HREZ daily regimen by local physician but she could not tolerate the drugs and developed jaundice after about 2 weeks; so, she discontinued the ATD on her own. After consulting a second physician, she took HREZ regimen for 2 months but the size of the neck nodes were increasing, and she discontinued the ATD on her own. After 1 month, she attended our OPD for check-up. She had no significant past history. At the time of presentation, she had significant enlargement of cervical lymph nodes with abscess formation. The patient had pallor, tachycardia, tachypnoea, and fever. Blood examinations were normal, and the patient was immunocompetent. Abscess was aspirated, and pus examination revealed the presence of AFB but Gram stain, fungal stain, and pyogenic culture were negative. Pus was sent for culture of *Mycobacterium tuberculosis* complex (MTB) by BACTEC method. CT scan of neck and chest showed cervical and mediastinal lymphadenopathy with central necrosis. Excision biopsy of the cervical lymph node revealed only necrotic tissues. After 8 weeks, pus culture showed growth of *Mycobacterium tuberculosis* complex which was resistant to isoniazid and rifampicin. So, the second-line ATD (kanamycin, ethionamide, levofloxacin, ethambutol, pyrazinamide, and cycloserine) was started. The patient tolerated the drugs well and sizes of the lymph nodes decreased significantly during follow-up at 6 months. The patient completed the treatment of twenty-four months successfully.

### Case 4

**Figure 3.**
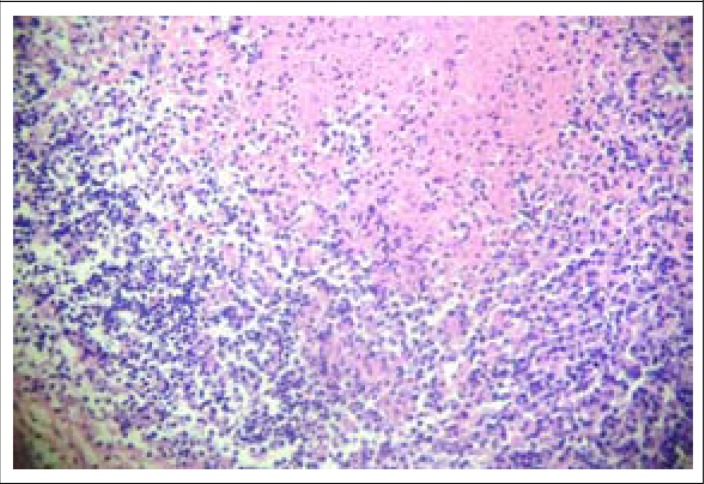
Histopathological picture of left cervical lymph node showing evidence of Kikuchi's disease (H&E stain, ×400)

A twenty-two years old girl presented with gradually increasing left-sided neck swelling for 2 months. FNAC from the lymph node revealed necrosis only, without any AFB. Despite that picture, family physician started antitubercular treatment with 2HREZ/4HR. When we examined, the swelling was tender, mobile, without any sinus or fever. Clinical examination did not reveal any abnormality. Her routine blood examination, chest x-ray, mantoux test, HIV reports, and connective tissue profile were normal. Excision biopsy of the left cervical lymph node revealed lymphoid follicles with germinal centres. Areas of necrosis containing karyorrhectic debris were present. Necrotic areas were surrounded by histiocytes, lymphocytosis, and plasma cells. Entire picture is suggestive of Kikuchi's disease (Figure 3). Z-N stain and *Mycobacterium tuberculosis* culture (MGIT 960) of the biopsy tissue were negative. The patient was better without any specific therapy.

## DISCUSSION

Tuberculous involvement of extra-pulmonary sites occurs in 15-20% of all cases of tuberculosis in India (1). Lymph nodes are the most common site for the extra-pulmonary tuberculosis, and it is most commonly-seen in children (1). Maharjan *et al.* showed that 54% of cervical lymphadenopathy is due to tuberculosis, 33% due to reactive lymphadenitis, and 11% cases due to metastases (3). In India, tuberculous lymphadenitis accounts for 35% of cases (1). Hence, even in India (located in TB-endemic zone), more than 50% cases of peripheral lymphadenopathy is due to aetiologies other than tuberculosis and, in these cases, excision biopsy with histopathology and microbiological examination is the only way to exclude tuberculosis. Although in resource-poor countries, like India, this type of invasive procedure in initial evaluation of lymphadenopathy before starting antitubercular therapy (ATT) is not always possible in all cases but one should keep in mind that it may be essential in some cases where the TB diagnosis is not confirmed, supportive evidences and clinical background are poor, and in non-responders to ATD. Clinical evidences, like systemic symptoms, discharging sinuses, matting, positive tuberculin skin tests, and associated pulmonary involvement by TB bacilli, are suggestive of tuberculous involvement of lymph nodes. Caseation and demonstration of acid fast bacilli on cytology are almost always suggestive of tuberculous aetiology but problems arise when cytological evidences are inconclusive (for example, poorly-formed granuloma, neutrophilic infiltration, absence of acid fast bailli, etc.) and also in non-responders after initial ATD therapy, where drug resistance and paradoxical reactions are two common reasons. Besides lymphomas, both Hodgkin disease and non-Hodgkin lymphoma (NHL) may be associated with immunodeficiency, which may lead to development of tuberculosis. Hence, lymphoma and TB can co-exist in the same patient, and in initial treatment failure of either lymphoma or TB, co-existence of both the diseases should be ruled out (4-6). On the other hand, immunosuppressive chemotherapy in lymphoma may help develop TB disease due to immunodeficiency, or worsen the clinical course of TB (7,8). The granuloma may occur in regional and/or distant lymph nodes or within the tumour itself (9). Non-caseating epitheloid granuloma and caseous necrosis both are found in tuberculosis (10). In lymphoma, development of granulomas may be due to immune or inflammatory reaction to tumour-associated antigenic determinants (11), or to the production of cytokines by the tumour cells (10), especially in Hodgkin disease. The diagnosis of TB and lymphoma may be difficult due to similarities in the clinical course, laboratory tests, and imaging procedures. In such cases, excision biopsy is the only way to differentiate them. Core biopsy is 96% sensitive and 100% specific in diagnosis of lymphoma (12). Kikuchi-Fujimoto disease (KFD) is histiocytic necrotizing lymphadenitis, first described by Dr Masahiro Kikuchi in 1972 in Japan. It is a rare, self-limiting disorder that typically affects the cervical lymph nodes (13), mimicking tuberculosis. It mainly occurs in young adults, with female preponderance. It is thought that KFD is a non-specific hyper-immune reaction to a variety of infectious (mainly viral), chemical, physical and neoplastic agents (14). The diagnosis of KFD is only established after histopathological examination.

In non-responders after initial ATT intake, drug resistance should be ruled out. Hence, mycobacterial culture and drug sensitivity test of the biopsy specimen should be done to exclude drug resistance. However, paradoxical reaction, HIV infection, uncontrolled diabetes, etc. are other causes for therapy failure, which should be considered in differential diagnosis. In our report, all the four patients were receiving ATT either empirically or on the basis of inconclusive cytology but the histopathological examination of the biopsy material proved the specific aetiology.

### Conclusions

In the clinical setting of cervical lymphadenopathy, not only tuberculosis but other causes, like lymphoma and HIV infection, should be considered (15). Especially when the lymphadenopathy is not responding to the adequate regimen of ATD, excision biopsy should be considered without hesitation. This consideration is also important because lymphoma is associated with cellular immunity suppression, predisposing the subject to tuberculosis (4). In this case series, it is obvious that excision biopsy is very much useful in diagnosing the aetiology of lymphadenopathy, especially in cases of non-responders.

## References

[1] Sharma SK, Mohan A (2004). Extrapulmonary tuberculosis. Indian J Med Res.

[2] Zeka AN, Tasbakan S, Cavusoglu C (2011). Evaluation of the Genexpert MTB/RIF Assay for rapid diagnosis of tuberculosis and detection of rifampin resistance in pulmonary and extrapulmonary specimens. J Clin Microbiol.

[3] Maharjan M, Hirachan S, Kafle PK, Bista M, Shrestha S, Toran K (2009). Incidence of tuberculosis in enlarged neck nodes, our experience. Kathmandu Univ Med J (KUMJ).

[4] Ouedraogo M, Ouedraogo SM, Cisse R, Lougue C, Badoum G, Sigani A (2000). [Active tuberculosis in a patient with Hodgkin's disease. A case report]. Rev Pneumol Clin.

[5] Audebert F, Schneidewind A, Hartmann P, Kullmann F, Schölmerich J (2006). [Lymph node tuberculosis as primary manifestation of Hodgkin's disease]. Med Klin (Munich).

[6] del Mar Bellido M, Martino R, Martìnez C, Sureda A, Brunet S (1995). Extrapulmonary tubercolosis and non-Hodgkin's lymphoma: coexistence in an abdominal lymph node. Haematologica.

[7] Smedby KE, Hjalgrim H, Askling J, Chang ET, Gregersen H, Porwit-MacDonald A (2006). Autoimmune and chronic inﬂammatory disorders and risk of non-Hodgkin lymphoma by subtype. J Natl Cancer Inst.

[8] Meya DB, McAdam KPWJ (2007). The TB pandemic: an old problem seeking new solutions. J Intern Med.

[9] Braylan RC, Long JC, Jaffe ES, Greco FA, Orr SL, Berard CW (1977). Malignant lymphoma obscured by concomitant extensive epithelioid granulomas: report of three cases with similar clinicopathologic features. Cancer.

[10] Hall PA, Kingston J, Stansfeld AG (1988). Extensive necrosis in malignant lymphoma with granulomatous reaction mimicking tuberculosis. Histopathology.

[11] Hollingsworth HC, Longo DL, Jaffe ES (1993). Small noncleaved cell lymphoma associated with florid epithelioid granulomatous response. A clinicopathologic study of seven patients. Am J Surg Pathol.

[12] Amador-Ortiz C, Chen L, Hassan A, Frater JL, Burack R, Nguyen T (2011). Combined core needle biopsy and fine-needle aspiration with ancillary studies correlate highly with traditional techniques in the diagnosis of nodal-based lymphoma. Am J Clin Pathol.

[13] Kikuchi M (1972). Lymphadenitis showing focal reticulum cell hyperplasia with nuclear debris and phagocytes. Acta Hematol Jpn.

[14] Bosch X, Guilabert A, Miquel R, Campo E (2004). Enigmatic Kikuchi-Fujimoto disease: a comprehensive review. Am J Clin Pathol.

[15] Ferrer R (1998). Lymphadenopathy: differential diagnosis and evaluation. Am Fam Physician.

